# Fabrication of Three-Dimensional Multilayer Structures of Single-Walled Carbon Nanotubes Based on the Plasmonic Carbonization

**DOI:** 10.3390/nano11092213

**Published:** 2021-08-27

**Authors:** Hao Cheng, Taeuk Lim, Hyunjoon Yoo, Jie Hu, Seonwoo Kang, Sunghoon Kim, Wonsuk Jung

**Affiliations:** 1 School of Mechanical Engineering, Chungnam National University, Daejeon 34134, Korea; chenghao@g.cnu.ac.kr (H.C.); taewook9409@g.cun.ac.kr (T.L.); uhz1996@o.cnu.ac.kr (H.Y.); h387669019@gmail.com (J.H.); sunwoo6752@naver.com (S.K.); 2 Department of Electronics Convergence Engineering, Wonkwang University, 460 Iksan-daero, Iksan 54538, Korea

**Keywords:** single-walled carbon nanotubes, 3D multilayer structure, plasmonic heating, carbonization, vacuum filtration, electromechanical stability

## Abstract

We developed a complex three-dimensional (3D) multilayer deposition method for the fabrication of single-walled carbon nanotubes (SWCNTs) using vacuum filtration and plasmonic carbonization without lithography and etching processes. Using this fabrication method, SWCNTs can be stacked to form complex 3D structures that have a large surface area relative to the unit volume compared to the single-plane structure of conventional SWCNTs. We characterized 3D multilayer SWCNT patterns using a surface optical profiler, Raman spectroscopy, sheet resistance, scanning electron microscopy, and contact angle measurements. Additionally, these carbon nanotube (CNT) patterns showed excellent mechanical stability even after elastic bending tests more than 1000 times at a radius of 2 mm.

## 1. Introduction

In recent decades, carbon nanotubes (CNTs) have become the focus of considerable research attention because of their extraordinary mechanical, electrical, and optical properties; they have become an ideal material in nanotechnology applications [1,2]. The special electrical properties of CNTs have paved the way for application in a series of flexible electronic devices, including transistors [3,4], sensors [5,6,7], photonics [8], electrodes [9,10], radio frequency identification tags [11], biological sensing labels [12,13], and many more devices [14]. In addition, their improved flexibility and reduced production costs have further consolidated their position as a candidate material in novel applications [15]. In order to use CNTs as electrical devices that perform different functions, their multiple layers and complex structures are still very challenging in terms of simplifying the manufacturing process.

Until now, CNTs have been patterned on different substrates to make devices, using processing technologies such as dip coating [16], inkjet printing [17,18], spray coating [19,20], chemical vapor deposition (CVD) catalyst patterning [21], plasma etching [22], laser irradiation [23], and electrodeposition [24]. However, in cases of dip coating and spray coating, only very small amounts of CNTs can be deposited in each layer of the coating. CVD catalyst patterning, plasma etching, and laser irradiation destroy the carbon nanotube sheets during the processing, thereby affecting the characteristics of the carbon nanotubes; and in cases of the electrodeposition process, the equipment required for production is expensive.

However, vacuum filtration [25,26] is a simple process that requires a very short time, provides great potential for mass production, and can achieve large-area uniform deposition of various 1D or 2D nanomaterials such as AgNW [27], CuNW [28], and graphene oxide [29]. Wu et al. [25] used a carbon nanotube film made by vacuum filtration to construct an electric-field-activated light modulator. The modulator can facilitate the optical simulation of field-effect transistors based on nanotubes. Alzaid, et al. [26] used hybrid films of carbon nanotubes and spherical nanocrystals made by vacuum filtration and enhanced the film’s elasticity using colloidal nanocrystal. However, in order for the CNT film to be patterned when using vacuum filtration, light masking is required. It is difficult to fabricate masks with complex shapes such as closed loops; moreover, a multilayer structure is not feasible. Thus, despite its various advantages, owing to these manufacturing difficulties, vacuum filtration has not been used in the deposition of complex multilayered CNT patterns.

In this paper, we propose a new method of patterning CNT films by assembling the basic pattern created by the existing vacuum filtration method and using the carbonization effect based on the plasma heating process to create complex multilayer shapes. The halogen lamp induces photon energy in the SWCNTs and subsequently generates carbonization, attributed to heat and a large number of carriers. The carbonization from the SWCNTs improves the mechanical stability of the three-dimensional multilayer structures. The shapes created in this way have unique 3D structures, which are different from those created with existing manufacturing methods; moreover, complicated photomasking is not required. Therefore, the manufacturing costs can be significantly improved. In addition, the 3D shape and the electrical and physical properties of the patterned CNTs film were also studied, and its excellent electrical and mechanical performance was verified through bending tests.

## 2. Experiments and Method

### 2.1. SWCNT Solution

Ultrasonic waves (using KFS-250n (Korea Process Technology Co., Ltd., Seoul, Korea) with an output power of 150 W) and surfactant SDS (sodium dodecyl sulfate, SIGMA Aldrich (Thermo Fisher Scientific Korea Ltd., Seoul, Korea), ACS reagent ≥ 99.0%) were used to uniformly disperse the single-walled carbon nanotubes (SWCNTs) (TUBALL, diameter < 2 nm, length > 1 μm, SWCNTs: 76%, metal impurities ≤ 15%) in the vacuum filtration solution [30]. First, 0.5 g (1 wt%) of SDS was added to 50 mL of deionized (DI) water and ultrasonicated (30 W) to dissolve and disperse for 10 min, after which 15 mg of SWCNTs was added to the solution and ultrasonicated (90 W) for 30 min to disperse the solution again. Finally, after 20 μL of SWCNTs was added, the solution was dispersed in DI water for the vacuum filtration process.

### 2.2. PDMS Molds and Fabrication of 3D Multilayer Pattern of SWCNTs

A polydimethylsiloxane (PDMS) mold was manufactured by casting using 3D printing. To complete the basic casting modeling, a 3D design program (Solid Works, SOLIDWORKS 2016 x16 Edition, Dassault Systemes Solidworks Corporation, Seoul, Korea) and 3D printer (Ultimate 2+) were used. After mixing the silicone elastomer (SYLGARD 184) base material and the curing agent at a ratio of 10:1 and stirring with a glass rod for 15 min, the mixture was degassed in a vacuum for 2 h to eliminate bubbles. In order to prevent the pattern from being damaged when the PDMS was removed, a urethane release agent was sprayed in advance, and the resulting solution was poured into the casting and heated in an oven at 60 °C for 4 h. The finished mold was applied to the vacuum filtration system (Supplementary Figure S1).

**Figure 1:**
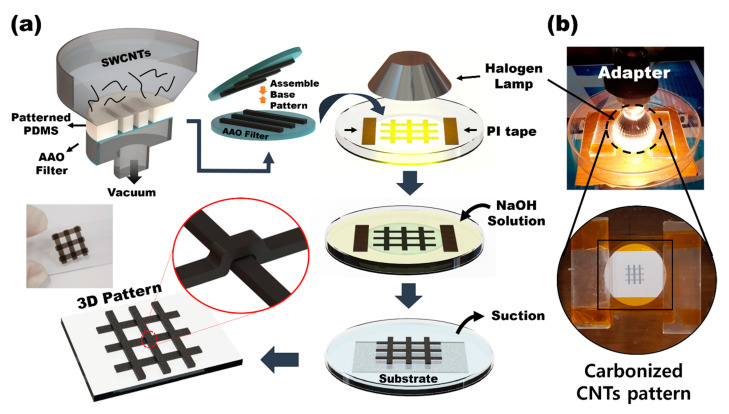
(**a**) Schematic diagram of the multiple steps of vacuum filtration to make base CNT patterns and assembling 3D patterns; PI tape is used for alignment and contact. (**b**) Induction of carbon welding using halogen lamps.

The 3D pattern was produced by combining the basic patterns made by vacuum filtration (Figure 1). The basic PDMS mold was fixed on an anodized aluminum oxide (AAO) filter, and the CNT solution was poured into the vacuum filtration barrel, as shown in Figure 1. The basic patterns were created at right angles to each other and fixed with polyimide (PI) tape for alignment and contact; the induction of carbon welding was done using halogen lamps [13]. The halogen lamp power was 35 W, the processing time was 1 h, and the distance from the substrate to the lamp was 4 mm. After filtering, the filter was dissolved in 10% NaOH solution (6 h) and washed 1–2 times with deionized (DI) water (over 1 h). This solution was replaced using a pump with an aspirator (Eyela, A-1000S SUNIL EYELA CO., LTD., Incheon, Korea). Finally, the completed pattern was transferred to the substrate in DI water, and the water was removed at room temperature. It has been reported that crating patterns are stretchable and have high mechanical stability [31,32,33].

Compared to the conventional fabrication method of making patterns of CNTs, we propose a simple fabrication process that can deposit complex multilayer SWCNT-based structures, as shown in Figure S2. In the conventional photolithography method, it is necessary to perform photoresist (PR) coating on the CNTs, etch them using O_2_ plasma to create patterns, and PR strip. These processes induce removing CNTs with PR, leaving residues of PR on the CNT surface, and preventing the 3D multilayer structure of CNTs because of process limitations. As shown in Figure S2, a single-layer pattern of SWCNTs can be deposited using the conventional fabrication method with O_2_ plasma. However, it is not possible to stack complex multilayers, such as two intersecting lines, as shown in Figure S2. Nevertheless, when decomposing a multilayer pattern, the conventional methods need complex processes and subsequently cause problems similar to those mentioned above. Conversely, using the novel fabrication process presented in this paper, we can verify that a complex multilayer can be constructed, as shown in Figure 1.

## 3. Results and Discussion

### 3.1. Analysis of 3D Fabrication of Complex Multilayer SWCNTs

**Figure 2:**
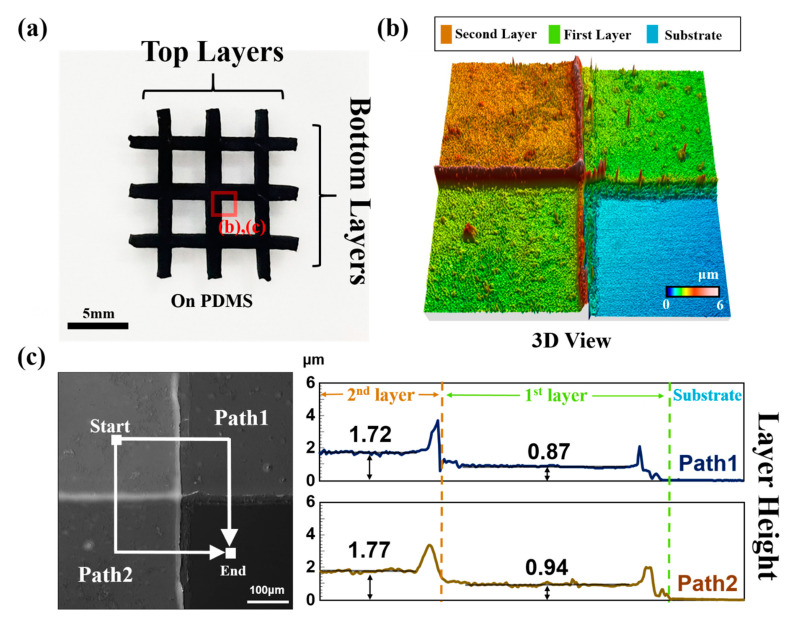
Three-dimensional (3D) analysis of a 3D SWCNTs pattern. (**a**) 3D SWCNT pattern on PDMS. (**b**) Optical surface profiler topographic image of a part of the pattern. (**c**) Vertical profile of a part of the pattern.

The complex multilayer CNT structure fabricated by the previous fabrication process is shown in Figure 2a. Three lines form the first layer; three lines in the second layer are deposited and crossed over three lines in the first layer sequentially. The CNT pattern during the DI water flushing process is shown in Figure 2a. By combining basic patterns, various omnidirectional patterns and rectangular grid shapes can be generated.

To clearly distinguish each layer, we used an optical surface profiler (Nanofocus AG, m-surf (NanoFocus AG, Oberhausen, Germany)) to analyze the surface shape of the sample. The intersecting point of the three lines was analyzed. Each layer could be distinguished separately; it was observed that the layers were packed in a dense and linear manner. This was owing to the characteristics of the vacuum filtration process that guarantees the uniformity of the film [25,34,35]. In addition, there were relatively clear edges for the following reasons: 1. During the filtration process, the vacuum pressure improved the adsorption force between the PDMS mask and the filter. 2. The vertically arranged micropores in the AAO mask limited the CNTs (cake filtration was performed because the length of the CNTs was larger than the micro-pore) [36]. The grid border and pattern edge were of uneven height because of the sliding of the CNTs when the PDMS mold was removed after filtering or during the bonding of the AAO mold [34].

Through the analysis of the vertical structure, it was verified that there was no damage to the thickness of each layer after they were stacked. The results are shown in Figure 2c. When the thickness of the first layer and the thickness of the second layer were compared, it was observed that the thickness of the second layer was not reduced (+1.5–1.15%). This was possible because all the patterns were carbonized on the interface without mechanical force. In addition, both Path1 and Path2 showed uniform height values for each layer, as in Figure 2. However, the limit resolution of the deposited CNT line needs to be validated by subsequent studies.

### 3.2. Numerical Analysis of the 3D Pattern

The advantage of a 3D pattern over 2D is that it has a larger specific surface area (SSA) in a limited space. In addition, in case of 2D patterns, as the number of layers increase, the manufacturing time grows, and the efficiency becomes low. Using the carbonization effect in this study, 3D patterned CNTs can be manufactured faster by simply stacking layers by layers; moreover, it is possible to construct a CNT with a larger specific surface area.

**Figure 3:**
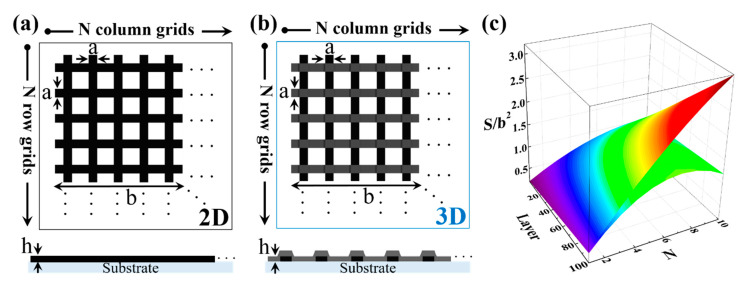
Numerical analysis of 3D pattern. (**a**) 2D model of pattern. (**b**) 3D model of pattern. (**c**) Specific surface area of 2D and 3D.

To analyze the 3D size effect more effectively, a numerical approach is proposed. For more realistic models, the shapes shown in Figure 3a,b were modeled based on the shapes shown in Figure 2. Figure 3a presents a 2D model, whereas a 3D model is presented in Figure 3b. Numerical processing was performed using MATLAB (Matlab R2017a, Mathworks Korea Co., Ltd., Seoul, Korea). The width was set to a, the length to b, and the height of a layer to h; they formed an N × N grid. As shown in Figure 3c, the number of layers and the parameters of the single grid increased, and the 3D model had a higher specific surface area than the 2D model. When using the newly proposed fabrication method, the number of stacked layers increased, and there was a very large surface area benefit compared to the conventional single-layer fabrication.

### 3.3. Membrane Characterization of Complex 3D Multilayer SWCNTs

**Figure 4:**
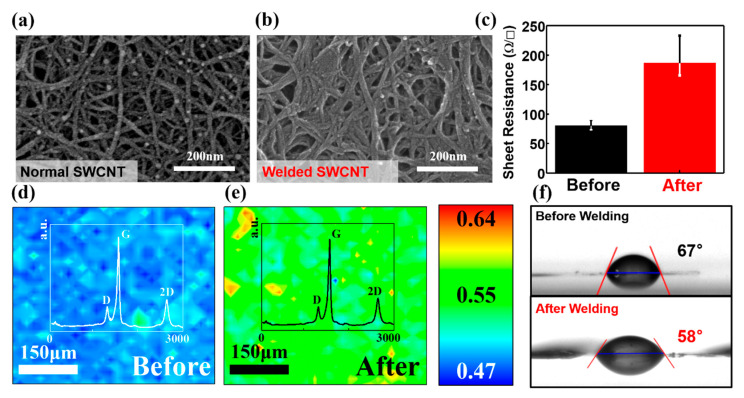
Microstructure of SWCNT 3D pattern. SEM image of SWCNT 3D pattern (**a**) before welding and (**b**) after welding. (**c**) Sheet resistance of 3D pattern before welding and after welding. Raman spectroscopy of 3D pattern (**d**) before welding and (**e**) after welding. (**f**) Contact angle measurement of 3D pattern before welding and after welding.

To verify the cause of the strong bonding force at the intersection of multiple layers, scanning electron microscopy (SEM) and Raman spectroscopy experiments were performed and the results analyzed. The microstructure of the sample was analyzed using FE-SEM (Hitachi, SU8230, Hitachi High-Technologies Corp., Tokyo, Japan). It was observed that only the filtered SWCNT pattern was individualized, the “hierarchical CNT” could be distinguished, and the cross-link was also confirmed, as shown in Figure 4a,b. After the halogen treatment, the cross-linking disappeared, and the surface became flat. Compared with the SWCNTs that were only filtered, in the SWCNTs designed here, the gap and area in the pattern were effectively reduced; the effect was similar to the trend of carbonization deformation [13]. The reasons for the similar carbonization effect in patterning are as follows: first, the halogen lamp was heated by radiant heat, mainly infrared and visible light radiation; then, even in a state of random arrangement, the SWCNTs were at a wavelength of 0.2–14 μm. This halogen lamp system induces photon energy at the SWCNT area by the inverse square law of the photons, which were highly concentrated on the center of the SWCNT sample [37,38]. The SWCNTs have excellent absorption characteristics [39], especially in the near-infrared spectroscopy (NIRs) region with special photoluminescence characteristics [40]. Therefore, surface plasmonic resonance (SPR) will be generated between the layered surfaces of the film by the part of the linearly polarized incident light propagating in the CNT fiber [37,38,41,42,43]; this results in the generation of heat and a large number of carriers. Moreover, the residual metal catalyst FeCO_3_ in the SWCNT powder decomposes at 280–490 °C: FeCO_3_ = Fe_3_O_4_ + CO_2_ + CO, and the CO_2_ and CO produced at this time are decomposed into C + CO_2_ at 250 °C. Atom C was a prerequisite for the sample. In this study, CNT defects and additional carbon layers were generated. Further research is required to conduct more accurate theoretical study.

The sheet resistance was measured using four probe measurements (AIT, CMT-100S, AIT Co., Ltd., Gyeonggi-do, Korea) to check the resistance change after welding, as shown in Figure 4c. The measuring point at room temperature was part of a layer to eliminate the influence of the thickness. It was observed that the resistance of the sample treated with the halogen lamp increased. The resistance increased from 80.369 to 186.73 Ω/square (132.3%). It is believed that the appearance of defects caused an increase in resistance compared to pristine CNTs.

Raman spectroscopy (Jasco, NRS-3100, Jasco Inc., Tokyo, Japan, 532 nm laser) was performed to characterize the crystal structure change of the pattern after halogen lamp treatment. The results of Raman spectroscopy, as shown in Figure 4d,e, revealed the crystal disorder, chirality, chemical impurities, curvature, and semiconducting behavior of the SWCNTs. Mapping spectroscopy was used to perform mapping at 100× magnification of a 50 µm × 50 µm ratio of area at 5 µm intervals. Raman mapping of 10 × 10 points showed that the average value of ID/IG decreased from 0.22 to 0.197 after heating, which was consistent with the results of previous studies [44]. The healing effect (Sp2 combination instead of hexagon, not amorphous but defective) occurred here; the ID/IG ratio decreased but the resistance R increased. We believe that the sample was hydrophilic due to the appearance of defects. The use of an additional electrical device did not affect the sample.

The water contact angle was investigated by Surface Elecyro Optics (SEO), phoenix 10, to measure the change in the contact angle before and after the halogen lamp treatment. As shown in Figure 4f, the contact angle after welding was reduced, and the hydrophilicity of the sample was stronger than before. Water molecules attempted to carry out “dissociative adsorption” to the defect sites on the surface of the nanotubes, thereby making the sample more hydrophilic [45]. 

### 3.4. Electromechanical Property of the 3D Pattern

**Figure 5:**
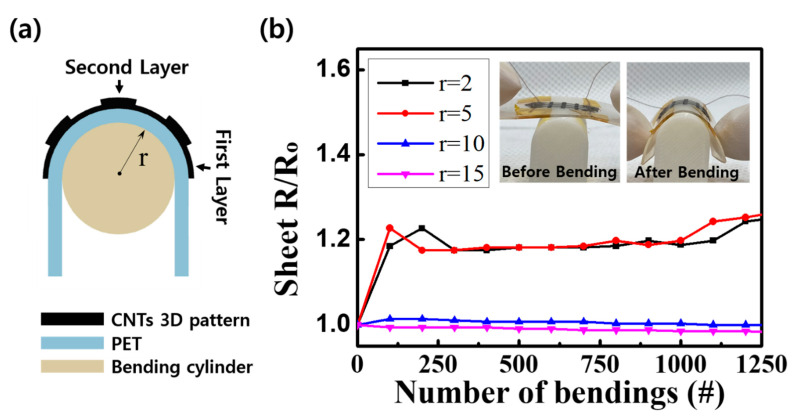
(**a**) Conceptual illustration of bending experiments applied to a CNT 3D pattern on the PET substrate. (**b**) Changes in the ratio of electrical sheet resistance to the initial resistance according to the number of bending experiments. An inset image shows the 3D pattern on the bending cylinder.

We also conducted a bending experiment to prove the electromechanical stability of the multilayer 3D SWCNT pattern samples manufactured by the newly proposed fabrication method using vacuum filtration and plasma welding processes [45]. The bending experiments were implemented through periodic and delicate bending motions of the SWCNTs on the PET (Polyethylene terephthalate) substrate, which were applied using cylinders with radii of 2, 5, 10, and 15 mm, as shown in Figure 5. The change in the sheet electrical resistance was measured for each bending motion using 2-point probe equipment (Keithley 2000, Tektronix, Inc., Seoul, Korea). Before the bending experiment began, the initial electrical sheet resistances were 0.309 kΩ at r (bending radius; r) = 2 mm, 0.255 kΩ at r = 5 mm, 0.285 kΩ at r = 2 mm, and 0.333 kΩ at r = 15 mm. The changes in the ratio of the electrical sheet resistance to the initial resistance of each 3D pattern are presented in Figure 5b. In the case of r = 2 mm and r = 5 mm, the electrical resistance sharply increased in the early stage of the bending test, which could be attributed to the onset of crack formation on the partial area of the CNT 3D pattern surface by the applied tensile force [45,46,47]. Subsequently, each electrical sheet resistance increased as the number of bending motions increased owing to the generation of additional cracks on other areas of the CNT 3D pattern by repetitive bending motions. These phenomena can be explained by the cracking, slipping, and tearing of the SWCNT layers [45,48,49,50]. In particular, when the number of bending cycles reached 1000, and in the case of r = 2 mm and r = 5 mm, the electrical resistance increased again as the number of bending cycles increased. This result could be attributed to several reasons such as lower adhesion energy between SWCNTs and the substrate, leading to less stretch during bending tests; spreading and releasing of folding and wrinkling on the 3D pattern surface were initially generated during the transfer processes before bending. In the case of r = 10 mm and r = 15 mm, the ratio of the sheet resistance change remained stable during the bending experiment. The initial sheet resistance values of the bending experiment with r = 10 mm and r = 15 mm were 0.285 and 0.333 kΩ, respectively. The sheet resistance values at the end were 0.284 and 0.327 kΩ, respectively, as shown in Figure 5b. These results showed that the 3D multilayers of the SWCNTs had excellent electromechanical stability, even after elastic bending tests. From these results we can conclude that the stability of the SWCNTs increased by several factors, such as the CNT networks comprising entangled dendritic bundles and competition between van der Waals adhesion and CNT bending effects [51] with the carbonization effect [37,38].

## 4. Conclusions

In summary, we developed a novel fabrication method based on vacuum filtration and PDMS mold transfer to construct multilayer 3D SWCNT structures. Highly increased mechanical durability and adhesion energy between stacked SWCNTs were achieved by welded junctions at the intersection of CNT lines using halogen lamps. SWCNTs can be stacked with complex multilayer structures in three dimensions using this fabrication method. These multilayer 3D SWCNT structures have a large surface area relative to the unit volume, compared to conventional single-layer patterns.

Furthermore, we characterized the multilayer SWCNTs through the analysis of an optical surface profiler, SEM, Raman spectroscopy, sheet resistance, and contact angle measurement. In addition, we observed excellent electromechanical stability even after elastic bending tests.

We anticipate that the conventional deposition method of SWCNTs can be replaced with this newly suggested fabrication method. If the PDMS mold has various shapes rather than one-dimensional lines, this suggested fabrication method can be used to fabricate more complex three-dimensional structures in addition to the one-dimensional and two-dimensional structures, which is expected to accelerate the research on wide-ranging applications in complex multilayer 3D structures of nanomaterials.

## Data Availability

Not applicable.

## Supplementary Materials

The following are available online at https://www.mdpi.com/article/10.3390/nano11092213/s1, Figure S1: PDMS Mold for 3D Pattern, Properties of SWCNTs, Figure S2: Process comparison of conventional photolithography method.

## References

[1] Aldalbahi A., Panhuis M.I.H. Inkjet printed conducting gel-carbon nanotube materials. Proceedings of the 2010 Conference on Optoelectronic and Microelectronic Materials and Devices (COMMAD).

[2] Small W.R., Panhuis M.I.H. (2007). Inkjet printing of transparent, electricallyconducting single-walled carbon-nanotube composites. Small.

[3] Beecher P., Servati P., Rozhin A., Colli A., Scardaci V., Pisana S., Hasan T., Flewitt A.J., Robertson J., Hsieh G.W. (2007). Ink-jet printing of carbon nanotube thin film transistors. J. Appl. Phys..

[4] Basiricò L., Cosseddu P., Fraboni B., Bonfiglio A. (2011). Inkjet printing oftransparent, flexible, organic transistors. Thin. Solid Films.

[5] Li J., Lu Y., Ye Q., Cinke M., Han J., Meyyappan M. (2003). Carbon nanotube sensorsfor gas and organic vapor detection. Nano Lett..

[6] Chopra S., Mcguire K., Gothard N., Rao A.M., Pham A. (2003). Selective gas detection using a carbon nanotubesensor. Appl. Phys. Lett..

[7] Zhang T., Mubeen S., Myung N.V.V. (2008). Recent progress in carbonnanotube-based gas sensors. Nanotechnology.

[8] Samanta S., Saini D., Singha A., Das K., Bandaru P.R., Rao A.M., Raychaudhuri A.K. (2016). Photoresponse of a single Y-junction carbon nanotube. ACS Appl. Mater. Interfaces.

[9] Liu X.M., Huang Z.D., Oh S.W., Zhang B., Ma P.C., Yuen M.M.F., Kim J.K. (2012). Carbon nanotube (CNT)-based composites as electrode material for rechargeableLi-ion batteries: A review. Compos. Sci. Technol..

[10] Kim C.L., Jung C.W., Oh Y.J., Kim D.E. (2017). A highly flexible transparent conductive electrode based on nanomaterials. NPG Asia Mater..

[11] Yang L., Zhang R., Staiculescu D., Wong C.P., Tentzeris M.M. (2009). A novel conformal RFID-enabled module utilizing inkjet-printed antennas andcarbon nanotubes for gas-detection applications. IEEE Antennas Wirel. Propag. Lett..

[12] Abera A., Choi J. (2010). Quantitative lateral flow immunosensor using carbonnanotubes as label. Anal. Methods.

[13] Kim J., Kim G.G., Kim S., Jung W. (2016). Plasmonic welded single walled carbon nanotubes on monolayer graphene for sensing target protein. Appl. Phys. Lett..

[14] Zhou Y.X., Hu L.B., Grüner G. (2006). A method of printing carbon nanotube thin films. Appl. Phys. Lett..

[15] Alshammari A.S., Alenezi M.R., Lai K.T., Silva S.R.P. (2017). Inkjet printing ofpolymer functionalized CNT gas sensor with enhanced sensing properties. Mater. Lett..

[16] Andrew Ng M.H., Hartadi L.T., Tan H., Patrick Poa C.H. (2008). Efficient coating oftransparent and conductive carbon nanotube thin films on plasticsubstrates. Nanotechnology.

[17] Kordas K., Mustonen T., Toth G., Jantunen H., Lajunen M., Soldano C., Talapatra S., Kar S., Vajtai R., Ajayan P.M. (2006). Inkjet Printing of Electrically Conductive Patterns of Carbon Nanotubes. Small.

[18] Tortorich R.P., Choi J.W. (2013). Inkjet Printing of Carbon Nanotubes. Nanomaterials.

[19] Kim S., Yim J., Wang X., Bradley D.D.C., Lee S., DeMello J.C. (2010). Spin-and spray-deposited single-walled carbon-nanotube electrodes for organic solar cells. Adv. Funct. Mater..

[20] Zhao X., Chu B.T.T., Ballesteros B., Wang W., Johnston C., Sykes J.M., Grant P.S. (2009). Spray deposition of steam treated and functionalized single-walledand multi-walled carbon nanotube films for super capacitors. Nanotechnology.

[21] Hofmann S., Cantoro M., Kaempgen M., Kang D.J., Golovko V.B., Li H.W., Yang Z., Geng J., Huck W.T.S., Johnson B.F.G. (2005). Catalyst patterning methods for surface-bound chemical vapor deposition of carbon nanotubes. Appl. Phys. A.

[22] Liu Y.M., Liu L., Liu P., Sheng L.M., Fan S.S. (2004). Plasma etching carbon nanotube arrays and the field emission properties. Diam. Relat. Mater..

[23] Kichambare P.D., Chena L.C., Wang C.T., Ma K.J., Wu C.T., Chen K.H. (2001). Laser irradiation of carbon nanotubes. Mater. Chem. Phys..

[24] Jeon Y.S., Byun J.Y., Oh T.S. (2008). Electrodeposition and mechanical properties of Ni–carbon nanotube nanocomposite coatings. J. Phys. Chem. Solids.

[25] Wu Z., Chen Z., Du X., Logan J.M., Sippel J., Nikolou M., Kamaras K., Reynolds J.R., Tanner D.B., Hebard A.F. (2004). Transparent, Conductive Carbon Nanotube Films. Science.

[26] Alzaid M., Roth J., Wang Y., Almutairi E., Brown S.L., Dumitrica T., Hobbie E.K. (2017). Enhancing the Elasticity of Ultrathin Single-Wall Carbon Nanotube Films with Colloidal Nanocrystals. Langmuir.

[27] Xu W., Xu Q., Huang Q., Tan R., Shen W., Song W. (2016). Fabrication of Flexible Transparent Conductive Films with Silver Nanowire by Vacuum Filtration and PET Mold Transfer. J. Mater. Sci. Technol..

[28] Ravinder R.K., Pallavi J., Ambuja N., Samir K.M., Dipti G. (2020). Fabrication of Silver Nanowire/Polydimethylsiloxane Dry Electrodes by a Vacuum Filtration Method for Electrophysiological Signal Monitoring. ACS Omega.

[29] Tang B., Zhang L., Li R., Wu J., Hedhili M.N., Wang P. (2016). Are vacuum-filtrated reduced graphene oxide membranes symmetric?. Nanoscale.

[30] Jung W., Woo J.Y., Lee S.H., Kim D., Kim S., Han C.S. (2012). Evaluation of the individualization state in single-walled carbon nanotube solutions using absorption, Raman and photoluminescence spectroscopy. Meas. Sci. Technol..

[31] Park M., Im J., Shin M., Min Y., Park J., Cho H., Park S., Shim M.B., Jeon S., Chung D.Y. (2012). Highly stretchable electric circuits from a composite material of silver nanoparticles and elastomeric fibres. Nat. Nanotechnol..

[32] Chung H.J., Sulkin M.S., Kim J.S., Goudeseune C., Chao H.Y., Song J.W., Yang S.Y., Hsu Y.Y., Ghaffari R., Efimov I.R. (2014). Ultrathin, Stretchable, Multiplexing pH Sensor Arrays on Biomedical Devices With Demonstrations on Rabbit and Human Hearts Undergoing Ischemia. Adv. Healthc. Mater..

[33] Ko C.M., Stoykovich M.P., Song J., Malyarchuk V., Choi W.M., Yu C.J., Geddes J.B., Xiao J., Wang S., Huang Y. (2008). A hemispherical electronic eye camera based on compressible silicon optoelectronics. Nature.

[34] Song X., Liu S., Gan Z., Lv Q., Cao H., Yan H. (2009). Controllable fabrication of carbon nanotube-polymer hybrid thin film for strain sensing. Microelectron. Eng..

[35] Hu L., Hecht D.S., Gruner G. (2004). Percolation in Transparent and Conducting Carbon Nanotube Networks. Nano Lett..

[36] Yang M., Kim S.W., Zhang S., Park D.Y., Lee C.W., Ko Y.H., Yang H., Xiao Y., Chen G., Li M. (2018). Facile and highly efficient fabrication of robust Ag nanowire–elastomer composite electrodes with tailored electrical properties. J. Mater. Chem. C.

[37] Kim J., Lee J., Kim S., Jung W. (2016). Highly Increased Flow-Induced Power Generation on Plasmonically Carbonized Single-Walled Carbon Nanotube. ACS Appl. Mater. Interfaces.

[38] Lin H.Y., Tsai W.H., Tsao Y.C., Sheu B.C. (2007). Side-Polished Multimode Fiber Biosensor Based on Surface Plasmon Resonance with Halogen Light. Appl. Opt..

[39] Nicola F.D., Hines P., Crescenzi M.D., Motta N. (2017). Thin randomly aligned hierarchical carbon nanotube arrays as ultrablack metamaterials. Phys. Rev. B.

[40] Akizuki N., Aota S., Mouri S., Matsuda K., Miyauchi Y. (2015). Efficient near-infrared up-conversion photoluminescence in carbon nanotubes. Nat. Commun..

[41] Govorov A.O., Richardson H.H. (2007). Generating Heat with Metal Nanoparticles. Nano Today.

[42] Yang Z., Sun X., Chen X., Yong Z., Xu G., He R., An Z., Li Q., Peng H. (2011). Dependence of Structures and Properties of Carbon Nanotube Fibers on Heating Treatment. J. Mater. Chem..

[43] Jeet K., Jindal V.K., Bharadwaj L.M., Avasthi D.K., Dharamvir K. (2010). Damaged Carbon Nanotubes Get Healed by Ion Irradiation. J. Appl. Phys..

[44] Dresselhaus M.S., Jorio A., Filho A.G.S., Saito R. (2010). Defect characterization in graphene and carbon nanotubes using Raman spectroscopy. Phil. Trans. R. Soc. A.

[45] Kim J., Kim G.G., Kim S., Jung W. (2016). Highly Enhanced Electromechanical Stability of Large-Area Graphene with Increased Interfacial Adhesion Energy by Electrothermal-Direct Transfer for Transparent Electrodes. ACS Appl. Mater. Interfaces.

[46] Majee S., Song M., Zhang S.L., Zhan Z.B. (2016). Scalable inkjet printing of shear-exfoliated graphene transparent conductive films. Carbon.

[47] Chen X.D., Liu Z.B., Jiang W.S., Yan X.Q., Xing F., Wang P., Chen Y., Tian J.G. (2013). The selective transfer of patterned graphene. Sci. Rep..

[48] Lin L.Y., Kim D.E., Kim W.K., Jun S.C. (2011). Jun Friction and Wear Characteristics of Multi-layer Graphene Films Investigated by Atomic Force Microscopy. Surf. Coat. Technol..

[49] Kwak B.W., Choi Y.C., Lee B.S. (2015). Small Variations in the Sheet Resistance of Graphene Layers with Compressive and Tensile Bending. Phys. E.

[50] Chen X., Yi C., Ke C. (2015). Bending Stiffness and Interlayer Shear Modulus of Few-layer Graphene. Appl. Phys. Lett..

[51] Drozdov G., Ostanin I., Xu H., Wang Y., Dumitrică T., Grebenko A., Tsapenko A.P., Gladush Y., Ermolaev G., Volkov V.S. (2020). Densification of single-walled carbon nanotube films: Mesoscopic distinct element method simulations and experimental validation. J. Appl. Phys..

