# Supplementary material for: Fabrication of Three-Dimensional Multilayer Structures of Single-Walled Carbon Nanotubes Based on the Plasmonic Carbonization
Source: Nanomaterials (Basel). 2021 Aug 27;11(9):2213. doi: 10.3390/nano11092213 (PMC8468131; doi:10.3390/nano11092213)
Supplement: Supplementary file 1 [file nanomaterials-11-02213-s001.zip › nanomaterials-1315723-supplementary.pdf]

nanomaterials

Article

Fabrication of Three-Dimensional Multilayer Structures of Single-Walled Carbon Nanotubes Based on the Plasmonic Carbonization

Hao Cheng [1,†], Taeuk Lim [1,†], Hyunjoon Yoo [1,†], Jie Hu [1], Seonwoo Kang [1], Sunghoon Kim [2,*] and Wonsuk Jung [1,*]

[1] School of Mechanical Engineering, Chungnam National University, Daejeon 34134, Democratic People's Republic of Korea; chenghao@g.cnu.ac.kr (H.C.); taewook9409@g.cun.ac.kr (T.L.); uhz1996@o.cnu.ac.kr (H.Y.); h387669019@gmail.com (J.H.); sunwoo6752@naver.com (S.K.)

[2] Department of Electronics Convergence Engineering, Wonkwang University, 460 Iksan-daero, Iksan 54538, Democratic People's Republic of Korea

* Correspondence: kshoon@wku.ac.kr (S.K.); wonsuk81@cnu.ac.kr (W.J.); Tel.: +82-42-821-6647 (W.J.)

† Contributed equally to this work.

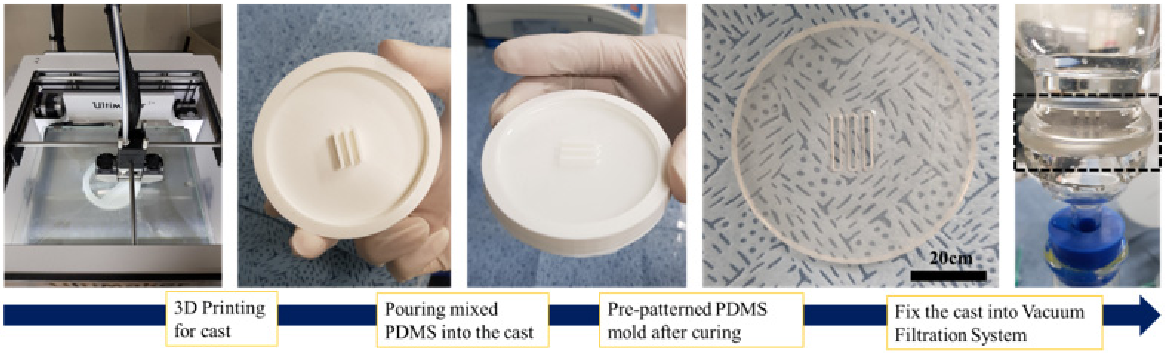

Figure S1. PDMS Mold for 3D Pattern.

Properties of SWCNTs

1. Physical Properties of CNTs

TUBALL, diameter: 1.6 ± 0.4 μm, length > 5 μm, SWCNTs ≥ 80 wt%, metal impurities ≤ 15 wt%, moisture < 5.

2. density of the Film

15 mg of CNTs was added to a mixture of 0.5 g and 50 mL of DI water, and then 20 μL of CNTs solution was added to DI water for vacuum filtration. One filtered solution contains about 0.00594 mg of CNT and the volume of a complete 3D pattern sample contains about 0.01188 mg of CNTs. The average thickness of a single layer in the 3D pattern is about 0.9 μm, and the length of a single CNTs film is 13 mm and the width is 1 mm. Therefore, the volume of a complete 3D pattern is calculated to be about 0.0117 mm^2 and the density of the film is about 1.0154 mg/mm^3.

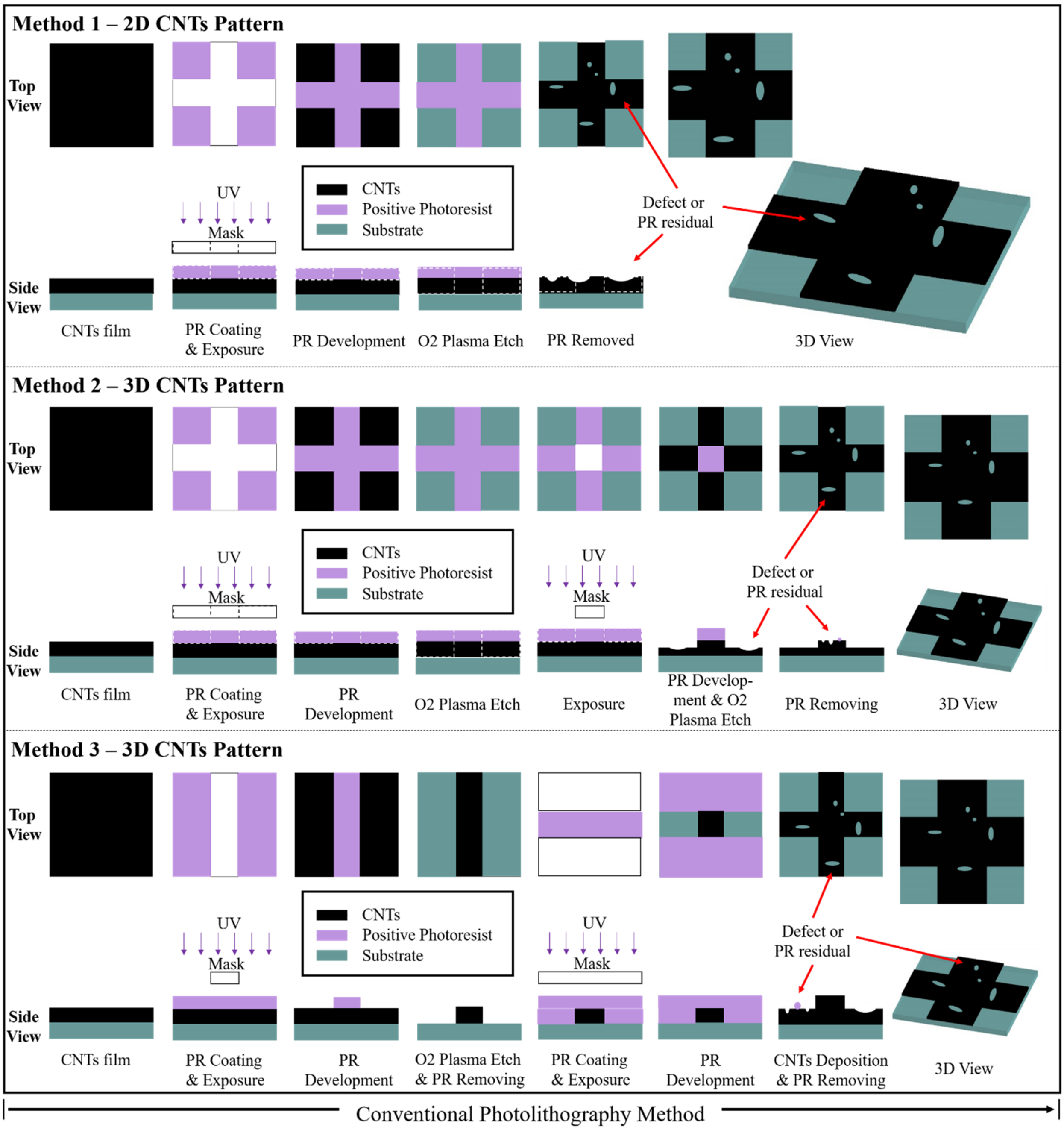

Figure S2. Process comparison of conventional photolithography method.
